# Live-cell imaging of endogenous CSB-mScarletI as a sensitive marker for DNA-damage-induced transcription stress

**DOI:** 10.1016/j.crmeth.2023.100674

**Published:** 2024-01-03

**Authors:** Di Zhou, Qing Yu, Roel C. Janssens, Jurgen A. Marteijn

**Affiliations:** 1 Department of Molecular Genetics, Oncode Institute, Erasmus MC Cancer Institute, Erasmus University Medical Center, Rotterdam, the Netherlands

**Keywords:** transcription-coupled repair, TC-NER, CSB, UVSSA, FRAP, nucleotide excision repair, DNA damage, transcription-blocking DNA damage

## Abstract

Transcription by RNA polymerase II (RNA Pol II) is crucial for cellular function, but DNA damage severely impedes this process. Thus far, transcription-blocking DNA lesions (TBLs) and their repair have been difficult to quantify in living cells. To overcome this, we generated, using CRISPR-Cas9-mediated gene editing, mScarletI-tagged Cockayne syndrome group B protein (CSB) and UV-stimulated scaffold protein A (UVSSA) knockin cells. These cells allowed us to study the binding dynamics of CSB and UVSSA to lesion-stalled RNA Pol II using fluorescence recovery after photobleaching (FRAP). We show that especially CSB mobility is a sensitive transcription stress marker at physiologically relevant DNA damage levels. Transcription-coupled nucleotide excision repair (TC-NER)-mediated repair can be assessed by studying CSB immobilization over time. Additionally, flow cytometry reveals the regulation of CSB protein levels by CRL4^CSA^-mediated ubiquitylation and deubiquitylation by USP7. This approach allows the sensitive detection of TBLs and their repair and the study of TC-NER complex assembly and stability in living cells.

## Highlights

• CRISPR-mediated, fluorescent tagging of endogenous TC-NER pathway proteins
• CSB mobility determined by FRAP is a sensitive marker for transcriptional stress
• CSB mobility measurements are a readout for TC-NER
• Flow cytometry of labeled proteins enables study of abundance changes after DNA damage

## Motivation

DNA damage that inhibits transcription and its repair has been thus far difficult to study in living cells. Therefore, using CRISPR-Cas9-mediated gene editing, we generated KI cells of the TC-NER proteins CSB and UVSSA. We show that measurement of especially CSB immobilization by fluorescence recovery after photobleaching (FRAP) is a sensitive marker to quantify transcription-blocking DNA damage and its repair.

## Graphical abstract

**Figure:**
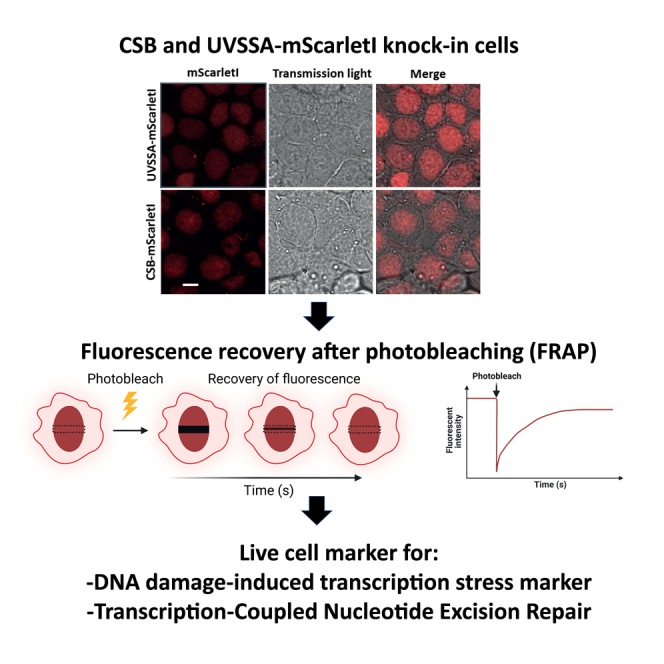

## Teaser

Zhou et al. generate cells with knockin fluorescent labeling of transcription-coupled repair proteins CSB and UVSSA. These tools enable fluorescence recovery after photobleaching (FRAP) studies to quantify transcription-blocking DNA damage and its repair in living cells.

## Introduction

RNA polymerase II (RNA Pol II) is responsible for the transcription of protein-coding genes in eukaryotic cells. Correct temporal and spatial regulation of RNA Pol II-mediated gene expression is crucial for proper cell function and tissue homeostasis. To safeguard this, transcription is tightly controlled at the different reaction steps of the transcription cycle, ranging from initiation, promoter-proximal pausing, and productive elongation to transcription termination.^1^^,^^2^ However, many DNA-damage-inducing agents from both environmental and endogenous origins pose a constant threat to the integrity of the DNA transcribed by RNA Pol II.^3^ Many of these DNA lesions, including UV- or cisplatin-induced DNA damage, can strongly impede or even completely block the progression of elongating RNA Pol II and are therefore referred to as transcription-blocking DNA lesions (TBLs).^4^^,^^5^^,^^6^ As a consequence, these TBLs cause an accumulation of lesion-stalled RNA Pol II, a lack of newly synthesized RNA molecules, or the onset of mutated mRNA.^7^ Consequently, if not correctly resolved, these TBLs will result in severe cellular dysfunction, apoptosis, or senescence, ultimately contributing to damage-induced aging.^7^^,^^8^^,^^9^^,^^10^

To overcome these severe implications, the highly efficient transcription-coupled nucleotide excision repair (TC-NER) pathway has evolved to specifically remove TBLs (Figure S1A). TC-NER is initiated by the recognition of lesion-stalled RNA Pol II by Cockayne syndrome group B protein (CSB). CSB can discriminate between lesion-stalled RNA Pol II and other non-forward-translocating RNA Pol II complexes, for example those stalled at natural pause sites.^11^ Using its ATP-dependent translocase activity, CSB pulls on the upstream DNA protruding from RNA Pol II, and as a consequence, it will push RNA Pol II forward over, e.g., natural pause sites. However, CSB cannot push RNA Pol II over bulky DNA lesions.^5^^,^^11^^,^^12^ As a consequence, the prolonged binding of CSB to lesion-stalled RNA Pol II is assumed to subsequently trigger the assembly of the full TC-NER complex consisting of CSA and UV-stimulated scaffold protein A (UVSSA). CSA forms together with DDB1, Rbx1, and Cul4A, a Cullin 4-RING-ubiquitin E3 ligase (CRL4) complex (CRL4^CSA^) in which CSA serves as the substrate recognition factor.^13^ The ubiquitylation activity of the CRL4^CSA^ E3 ligase is activated upon dissociation of the COP9 signalosome complex upon DNA damage.^13^ This subsequently results in the proteasomal degradation of CSB^13^^,^^14^^,^^15^ and, stimulated by ELOF1,^16^^,^^17^ in the ubiquitylation of lesion-stalled RNA Pol II at lysine 1268 of RPB1.^18^^,^^19^ The CRL4^CSA^-mediated CSB ubiquitylation is counteracted by the ubiquitin-specific protease 7 (USP7), which is recruited to lesion-stalled RNA Pol II by UVSSA, thereby stabilizing CSB during the TC-NER reaction.^20^^,^^21^^,^^22^ In addition to its interaction with USP7, UVSSA has an important role in recruiting the transcription factor II H (TFIIH) complex via its direct interaction with the TFIIH subunit p62.^18^^,^^23^^,^^24^ TFIIH stimulates the unwinding of 30 nucleotides around the DNA-damaged site by its xeroderma pigmentosum group B (XPB) and XPD subunits.^25^^,^^26^^,^^27^ With the assistance of XPA and replication protein A (RPA), TFIIH is responsible for the damage verification and the correct positioning of the XPF/excision-repair cross complementing-1 (ERCC1) and XPG endonucleases.^28^^,^^29^^,^^30^^,^^31^ Subsequently, the single-stranded gap generated by the excision of damaged DNA is filled by DNA synthesis and sealed by DNA ligase,^32^^,^^33^ after which transcription can restart.^34^

The significance of DNA-damage-induced transcription stress and functional TC-NER is clearly illustrated by the severe growth failure, photosensitivity, premature aging, and progressive neurodegenerative symptoms of CS, an inherited TC-NER-deficient human disorder caused by mutations in the CSA and CSB genes.^7^^,^^35^^,^^36^^,^^37^ Despite the biological relevance of DNA-damage-induced transcription stress, no sensitive live-cell imaging and quantitative analysis tools are currently available to detect RNA Pol II impediments by DNA damage. While RNA Pol II-mediated transcription is obstructed by TBLs, the effects on chromatin binding of GFP-tagged RNA Pol II as determined by fluorescence recovery after photobleaching (FRAP)^38^ were relatively mild, mainly due to the fact that only a subset of the total RNA Pol II will be arrested at a TBL, while other elongating RNA Pol II complexes are also tightly chromatin bound during the transcription process.^39^^,^^40^ In contrast, TC-NER factors are expected to specifically bind to lesion-stalled RNA Pol II complexes while not being bound to chromatin or RNA Pol II in unperturbed conditions.^16^^,^^24^ However, thus far, FRAP studies on fluorescent-tagged TC-NER factors like CSB^41^ or UVSSA^21^ have only resulted in very limited TBL-induced immobilizations, most likely caused by the exogenous overexpression of these TC-NER factors. Therefore, in this study, we generated fully functional mScarletI-tagged CSB and UVSSA knockin (KI) cells by CRISPR-Cas9-mediated gene targeting, which allows us to study the levels and dynamics of these proteins at their endogenous levels. We show that endogenously expressed mScarletI-tagged CSB combined with FRAP is a sensitive tool to detect DNA-damage-induced transcription stress in real time. The use of a dedicated set of TC-NER knockout (KO) cells allowed us to study the effect of these factors on mScarletI-CSB chromatin binding in living cells. Furthermore, these CSB-mScarletI KI cells allowed the efficient study of the effects of different TC-NER factors on protein levels of mScarletI-tagged CSB following DNA damage induction. Together, the developed TC-NER KI cells will provide insights into TC-NER complex assembly and composition and its dynamic regulation in the physiologically relevant environment of the living cell.

## Results

### Generation of CSB- and UVSSA-mScarletI KI cells

**Figure 1:**
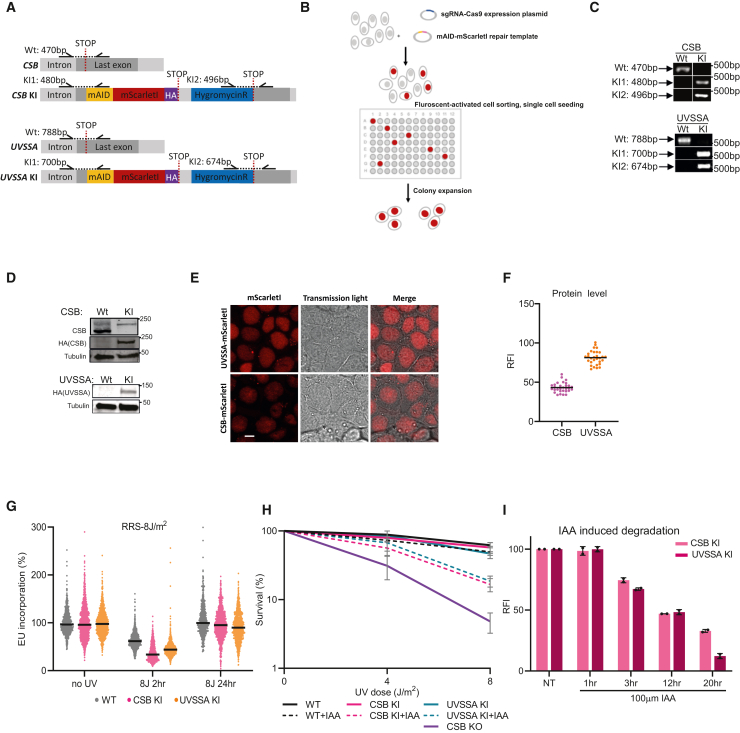
Generation of mAID-mScarletI-HA-tagged CSB and UVSSA knockin cells (A) Schematic view of the genomic locus of CSB and UVSSA and the used strategy for generating homozygous mAID-mScarletI-HA-tagged CSB and UVSSA knockin (KI) cell lines. Arrows indicate primer locations for PCR as depicted in (C). (B) Schematic representation of the procedure of CRISPR-Cas9-mediated gene editing to generate mAID-mScarletI-HA-tagged CSB and UVSSA KI HCT116 cells. A plasmid expressing the sgRNA and Cas9 was co-transfected with the indicated repair template. After hygromycin (100 μg/mL) selection for 7 days, FACS was used to sort mScarletI-positive cells as single cells into a 96-well plate. Single cells were expanded and subsequently collected for analysis. (C) Homozygous mScarletI-tagged CSB and UVSSA KI cells were confirmed by genotyping. Genomic DNA of HCT116 wild-type (WT) and KI cells was isolated and analyzed using PCR with the indicated primers (A and Key Resources Table). PCR products were analyzed by DNA gel electrophoresis. (D) Characterization of mScarletI-tagged CSB and UVSSA KI cells by immunoblot using the indicated antibodies. Tubulin was used as loading control. (E) Representative live-cell images of UVSSA- and CSB-mScarletI KI cells with fluorescent and transmission light images as indicated. Foci outside the nucleus are caused by autofluorescence, as these were also observed in WT cells. Scale bar: 10 μm. (F) Quantification of CSB- and UVSSA-mScarletI levels in living cells by confocal imaging. Background-corrected integrated nuclear intensity of CSB- and UVSSA-mScarletI signal is plotted per cell. Black lines indicate average integrated density of n = 30 cells from 2 independent experiments. RFI, relative fluorescence. (G) Transcription restart after UV-induced DNA damage as determined by relative 5-ethynyl uridine (EU) incorporation in HCT116 mScarletI-tagged CSB and UVSSA KI cells, 2 or 24 h after 8 J/m^2^ UV-C or mock treatment (non-treated [NT]). Relative integrated density of UV-irradiated samples is normalized to mock-treated cells and set to 100. Black lines indicate average integrated density of, respectively n = 667, 1,158, 877, 959, 1,001, 816, 585, 1,114, and 781 cells collected from 2 independent experiments. (H) Relative colony survival of HCT116 mScarletI-tagged CSB and UVSSA KI cells following exposure to indicated doses of UV-C, with or without 16 h pre-treatment of doxycycline (1 mg/mL) and auxin (0.5 mM). Plotted curves represent mean ± SD. n = 3. (I) mScarletI fluorescence levels as determined by flow cytometry analysis (FACS) of mScarletI-tagged CSB and UVSSA KI cells treated with doxycycline (1 mg/mL) and auxin (0.5 mM) for the indicated time points. Plotted bars represent average fluorescence of 2 independent experiments, ±SD.

To study the *in vivo* dynamics of endogenous expressed CSB and UVSSA, we generated cells expressing fluorescently labeled CSB and UVSSA proteins expressed from their endogenous locus. To do so, we made mini auxin-inducible degron (mAID)-mScarletI-hemagglutinin (HA)-tagged CSB and UVSSA KIs in HCT116 cells in a similar strategy to that previously described.^42^ The AID tag allows the swift degradation of the tagged protein. Upon auxin treatment, the AID-tagged protein bound by the exogenous expressed Oryza sativa F box transport inhibitor response 1 (OsTIR1) protein with the native Skp1–Cul1–F-box (SCF) E3 ubiquitin-ligase complex, resulting in the ubiquitylation of the target protein.^42^ We transiently expressed a single guide RNA (sgRNA) to induce a CRISPR-Cas9-mediated double-strand break (DSB) upstream of the stop codon of CSB or UVSSA. Repair templates containing mScarletI cDNA with a hygromycin B selection cassette flanked by homology arms of 200 base pairs comprised of genomic CSB or UVSSA sequences were co-transfected to allow repair of the DSB by homologous recombination (Figure 1A). Subsequently, cells were kept in culture medium containing 100 μg/mL hygromycin B for 7 days to enrich for cells that have successfully incorporated the mAID-mScarletI cassette (Figure 1B). Next, mScarletI-positive cells were isolated by fluorescence-activated cell sorting (FACS, and single-cell clones of CSB or UVSSA homozygous KI cells were selected, as shown by genotyping (Figure 1C). Immunoblot analysis showed that endogenously expressed mScarletI-tagged CSB and UVSSA are fully expressed (Figure 1D). Of note, CSB-mScarletI expression level status in KI cells was similar to wild-type (WT) cells. For UVSSA, such a direct comparison was not possible, as endogenously expressed UVSSA could not be detected by antibodies. Live cell imaging showed that UVSSA and CSB are expressed uniformly in the nucleus (Figure 1E) and that UVSSA is approximately 2-fold more highly expressed than CSB (Figures 1F and S1B). Importantly, mScarletI-tagged CSB and UVSSA showed a full RNA synthesis recovery (RRS) after UV damage, similar to the WT parental HCT116 cells (Figure 1G). Furthermore, colony survival of UVSSA- and CSB-mScarletI KI cells showed a similar UV sensitivity to WT cells, while CSB KO cells were UV hypersensitive (Figure 1H). Together, these experiments indicate that both UVSSA- and CSB-mScarlet fluorescent-tagged proteins are fully functional.

Next, we tested the degradation efficiency of the mAID tag in mScarletI-tagged CSB and UVSSA KI cells. Therefore, we monitored CSB and UVSSA protein levels by mScarletI fluorescence determined by flow cytometry analysis after the activation of the AID system by auxin and by doxycycline to induce OsTIR expression.^42^ CSB and UVSSA proteins were depleted, with a 50% reduction within 12 h of doxycycline/auxin addition (Figure 1I). Correspondingly, mScarletI-tagged CSB and UVSSA KI cells exhibited UV hypersensitivity upon depletion of CSB and UVSSA proteins, respectively (Figure 1H). However, doxycycline/auxin-induced CSB degradation led to milder UV sensitivity compared to CSB KO, which is most likely explained by the incomplete depletion of CSB and UVSSA proteins (Figure 1I). Together, these data show that in these KI cells, the mAID tag allows the swift degradation of CSB and UVSSA and that CSB and UVSSA protein levels can be easily assessed in living cells by their fluorescence intensities.

### CSB- and UVSSA-mScarletI KI cells as sensitive tools to detect UV-induced transcription stress

**Figure 2:**
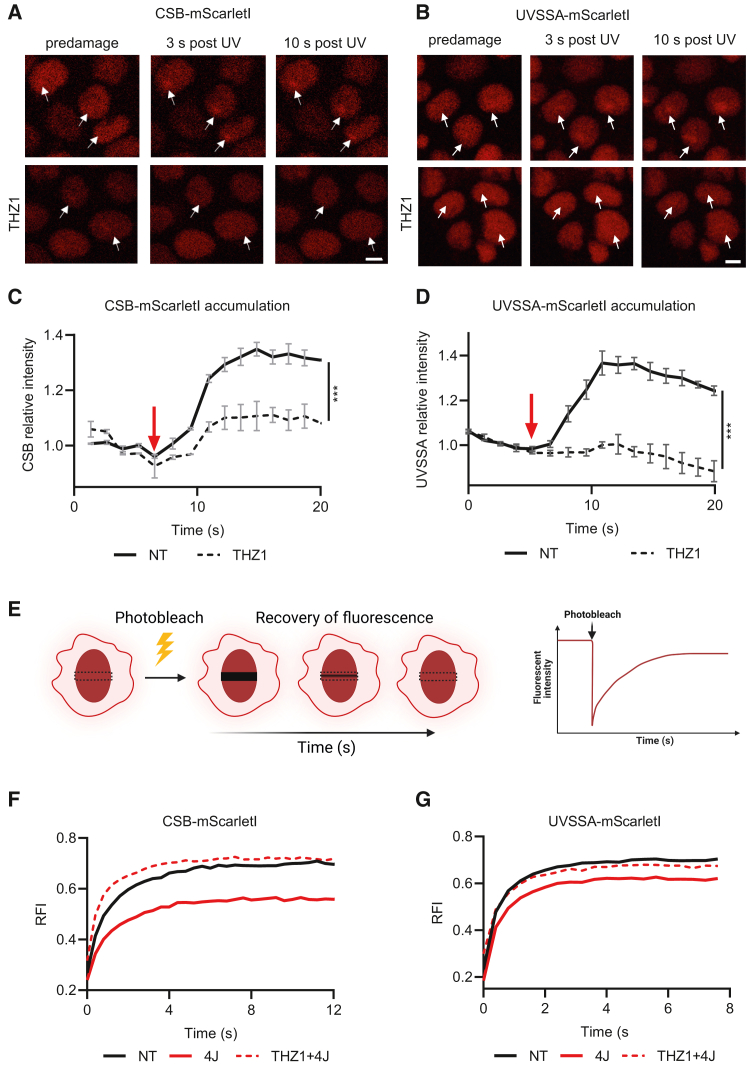
Transcription-dependent binding of CSB and UVSSA to damage chromatin (A and B) Representative images of mScarletI-tagged CSB (A) and UVSSA (B) KI cells upon local DNA damage induction with a UV-C (266 nm) laser. Where indicated, cells were pre-treated with the transcription inhibitor THZ1 (1 μM) for 90 min before damage induction. Arrows indicate site of DNA damage induction. Scale bar: 10 μm. (C and D) Relative accumulation of endogenously expressed mScarletI-tagged CSB (C) and UVSSA (D) at sites of local UV damage (LUD) induced with a UV-C (266 nm) laser. mScarletI fluorescence intensity at LUD was measured over time using live-cell confocal imaging and normalized to pre-damage intensity set at 1.0 at t = 0. The start of damage induction is indicated with a red arrow. Curves indicates average relative intensity of n > 37 cells in (C) and n > 42 cells in (D) (mean ± SD). Where indicated, cells were pre-treated with the transcription inhibitor THZ1 (1 μM) for 90 min before damage induction (dotted line). ∗∗∗p ≤ 0.001, area under the curve was analyzed by unpaired t test. (E) Schematic representation explaining the used FRAP procedure of mScarletI-tagged CSB and UVSSA cells in which a small strip within the nucleus is bleached by a high-intensity laser pulse, after which the recovery of fluorescence is measured over time. Cartoon created with BioRender.com. (F and G) FRAP analysis of mScarletI-tagged CSB (F) and UVSSA (G) KI cells in unperturbed conditions (NT) or within the first hour after UV-C (4 J/m^2^) irradiation. Where indicated, cells were pre-treated with the transcription inhibitor THZ1 (1 μM) for 90 min before UV irradiation (dotted line). mScarletI-tagged CSB and UVSSA were bleached and fluorescence intensity was measured every 0.4 s for 12 (CSB) or 8 s (UVSSA), background corrected, and normalized to pre-bleach fluorescence intensity (FI) set at 1.0. RFI, relative fluorescence intensity. Plotted curves represent the average of 3 (F) or 2 (G) independent experiments of 10 cells per experiment and condition.

Thus far, accumulation at local UV-induced DNA damage (LUD [local UV damage]) of exogenously expressed GFP-tagged UVSSA and CSB was used to study their activities during TC-NER.^21^^,^^41^^,^^43^^,^^44^ To confirm that the mScarletI-tagged CSB and UVSSA KI cells can be used to study TC-NER in living cells, we determined the accumulation kinetics of CSB and UVSSA to sites of LUD. We locally induced TBLs using a 266 nm UV-C laser^45^^,^^46^ and found a quick and modest accumulation at LUD (∼1.4-fold) of both endogenously expressed mScarletI-tagged CSB and UVSSA (Figures 2A–2D). As both TC-NER proteins are known to bind to lesion-stalled RNA Pol II,^21^^,^^41^^,^^43^^,^^44^ we next tested whether CSB and UVSSA are recruited to LUD in a transcription-dependent manner. To do so, we treated cells with the CDK7 inhibitor THZ1^47^ to block transcription initiation for 90 min before inducing LUD, thereby depleting elongating RNA Pol II before DNA damage induction.^39^ As expected, recruitment of both mScarletI-tagged CSB and UVSSA was significantly decreased upon inhibition of transcription, indicating that CSB and UVSSA accumulate at LUD in a transcription-dependent manner (Figures 2A–2D). The comparable accumulation kinetics of mScarletI-tagged CSB and UVSSA, in contrast to what has been observed for global genome-NER (GG-NER) factors,^21^ suggests a similar mode of recruitment to LUD for these TC-NER factors. Interestingly, despite the similarities of recruitment kinetics, which mostly provides information on the association constant (k_on_), CSB accumulation at LUD was more stable compared to UVSSA, as UVSSA showed a slight reduction at sites of LUD over time. This could indicate that UVSSA is shorter or more transiently bound at lesion-stalled RNA Pol II than CSB.

In addition to recruitment studies, we investigated the UV-induced chromatin binding of mScarletI-tagged CSB and UVSSA in TC-NER by FRAP analysis (Figure 2E). In contrast to accumulation at LUD, FRAP informs on the steady-state binding of proteins, which is influenced by both the association (k_on_) and dissociation (k_off_) constants of the protein of interest. Most GG-NER-involved factors, such as XPC, DDB2, and TFIIH, show significant reductions in protein mobility after UV irradiation, which reflects their engagement in repair.^48^^,^^49^^,^^50^^,^^51^ However, such DNA-damage-induced immobilization was very difficult to detect for exogenously expressed UVSSA and CSB.^21^^,^^41^

To test whether endogenously expressed mScarletI-tagged CSB and UVSSA can be accurately used to determine chromatin-binding kinetics in FRAP assays (Figure 2E), we investigated CSB and UVSSA chromatin binding following UV-induced DNA damage by FRAP. Shortly after UV irradiation at 4 J/m^2^, we detected a significant increase in immobilization of CSB (Figures 2G and S2; Videos S1 and S2) and UVSSA (Figures 2G and S3), indicating that CSB and UVSSA were more associated with chromatin upon DNA damage. The immobilization was also very reproducible between cells, as shown by the limited variation in the FRAP curves of single cells (Figures S4A–S4F; see Data S1 for all quantitative FRAP data of individual cells). Interestingly, this immobilization is more severe compared to the FRAP analysis with exogenously expressed GFP-tagged TC-NER proteins, which revealed a very minimal immobilization for CSB at a much higher UV dose (16 J/m^2^)^41^ or no immobilization at all for UVSSA.^21^ The clear UV-induced immobilization of endogenously expressed CSB and UVSSA shows the importance of performing these experiments at physiologically relevant expression levels, especially while studying protein mobility, where protein levels are expected to influence the experimental outcome. Importantly, the UV-induced immobilization of mScarletI-tagged CSB and UVSSA was fully dependent on transcription, as the transcription inhibitor THZ1 abolished the UV-induced immobilization of both proteins (Figures 2F and 2G). These data indicate that the observed transcription- and DNA-damage-dependent immobilization of CSB and UVSSA reflects their involvement in TC-NER.

Video S1. Movie of raw strip-FRAP images of untreated CSB-mScarletI cell, related to Figure 2

Video S2. Movie of raw strip-FRAP images of UV-irradiated (4 J/m^2^) CSB-mScarletI cell, related to Figure 2

**Figure 3:**
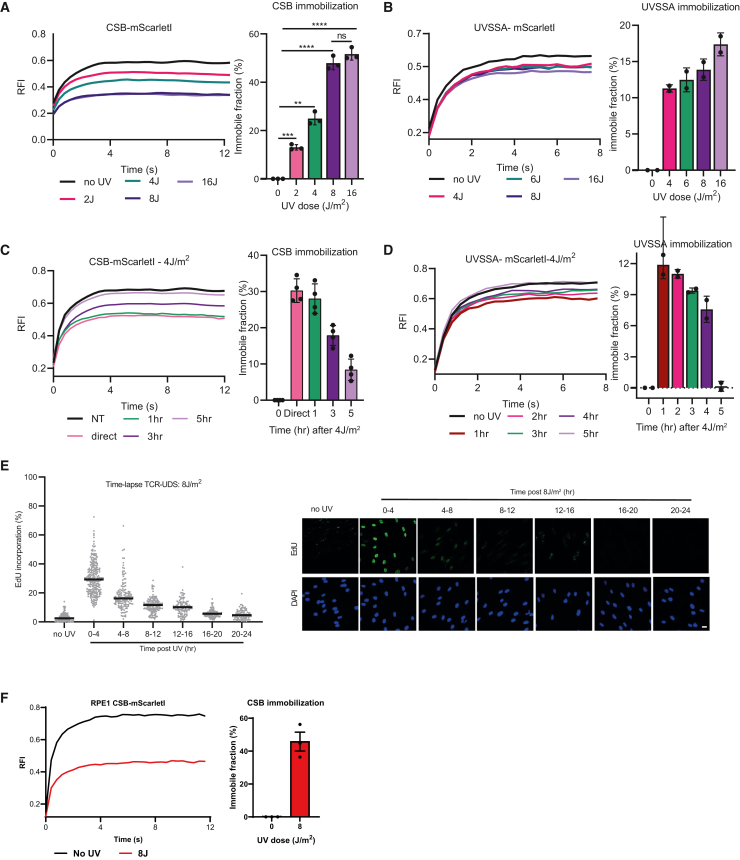
CSB and UVSSA chromatin-binding kinetics upon UV damage (A and B) FRAP analysis of mScarletI-tagged CSB (A) and UVSSA (B) in unperturbed conditions (no UV) or within the first 30 min after irradiation with the indicated UV doses. Curves represent average of 3 (A) or 2 (B) experiments of 10 cells each. Right: calculated immobile fractions of the depicted conditions from the left. Plotted values represent mean. Plotted curves represent the average ±SD of 3 independent experiments (A) or 2 independent experiments (B) of 10 cells per experiment and condition. ∗∗p ≤ 0.01, ∗∗∗p ≤ 0.001, ∗∗∗∗p ≤ 0.0001, analyzed by two-way ANOVA. (C and D) FRAP analysis of mScarletI-tagged CSB (C) and UVSSA (D) in unperturbed cells (NT) or after the indicated time points after UV-C irradiation (4 J/m^2^). Curves represent average of 3 (C) or 2 (D) experiments of 10 cells each. Right: calculated immobile fractions of the depicted conditions from the left. Plotted curves represent the average ±SD of 4 (C) or 2 (D) independent experiments of 10 cells per experiment and condition. (E) Quantification of the UDS mediated by TC-NER, as measured by the total nuclear fluorescence (Alexa Fluor 488 nm) at the indicated time slots in GG-NER-deficient XP186LV (XP-C) cells after EdU labeling for 4 h followed by tyramide signal amplification (TSA) signal amplification. Right: representative images of XP186LV (XP-C) cells irradiated with 8 J/m^2^ for the indicated time slots. Nuclei were identified by DAPI staining. Each dot represents a single cell, mean is shown by the black line, and >200 cells were collected in each condition. Scale bar: 30 μm. (F) FRAP analysis of mScarletI-tagged CSB in RPE1 cells in unperturbed conditions (no UV) or within the first 30 min after irradiation with 8 J/m^2^ UV. Curves represent average of 3 experiments of 10 cells each. Right: calculated immobile fractions of the depicted conditions from the left. Plotted curves represent the average ±SEM of 3 independent experiments of 8 cells per experiment and condition.

To study in detail the consequences of TBLs on CSB and UVSSA, we irradiated mScarletI-tagged CSB and UVSSA cells with increasing doses of UV and analyzed chromatin-binding kinetics of CSB and UVSSA by FRAP. Unlike comparable accumulation kinetics (Figures 2C and 2D), mScarletI-tagged CSB and UVSSA showed distinctive behavior in FRAP analysis. The UV-induced immobilization of CSB was much bigger and proportional to UV doses ranging from a ∼13% immobile fraction at 2 J/m^2^ to a plateau of ∼50% at 8 J/m^2^ (Figure 3A). This plateau of CSB immobilization at 8 J/m^2^ is in line with previous data showing that TC-NER activity saturates at a UV dose of 6–8 J/m^2^ (see Weinholz et al.^52^) as determined by TC-NER-dependent unscheduled DNA synthesis (TCR-UDS). UVSSA displayed a much smaller immobilization without a clear increase upon higher damage loads, e.g., increasing from ∼11% at 4 J/m^2^ to ∼14% at 8 J/m^2^ (Figure 3B).

TC-NER removes TBLs in time; therefore, we hypothesize that the immobilization of TC-NER factors will be reduced in time due to ongoing repair. We tested whether we could assess TC-NER activity in real time in living cells by assessing TBL-induced immobile fractions of CSB and UVSSA over time upon TBL induction. As expected, FRAP analysis of mScarletI-tagged CSB and UVSSA showed a time-resolved remobilization. This remobilization was almost comparable to untreated conditions after 4–5 h (Figures 3C and 3D), indicating that repair by TC-NER has been completed after 5 h. This is in line with our TCR-UDS data, which reveal that the majority of TC-NER, as determined by TC-NER-specific DNA repair synthesis,^52^ takes place in the first 4 h after UV irradiation (Figure 3E). Next, we tested whether this severe CSB immobilization was cell-type specific or whether similar results could be observed in different cell types. Therefore, we generated CSB-mScarletI KI cells in RPE1 cells using the same CRISPR-Cas9-mediated genome-editing approach as used for the CSB KI cells in HCT116. Irradiation of 8 J/m^2^ UV resulted in a similar CSB immobilization of approximately 50% (Figure 3F), in the same range as was observed in HCT116 cells. This indicates that severe CSB immobilization is a general response, and this approach furthermore shows the flexibility of this system to generate CSB KI cells in different cell types.

### CSB mobility as live-cell marker for different types of transcription-blocking DNA damage

**Figure 4:**
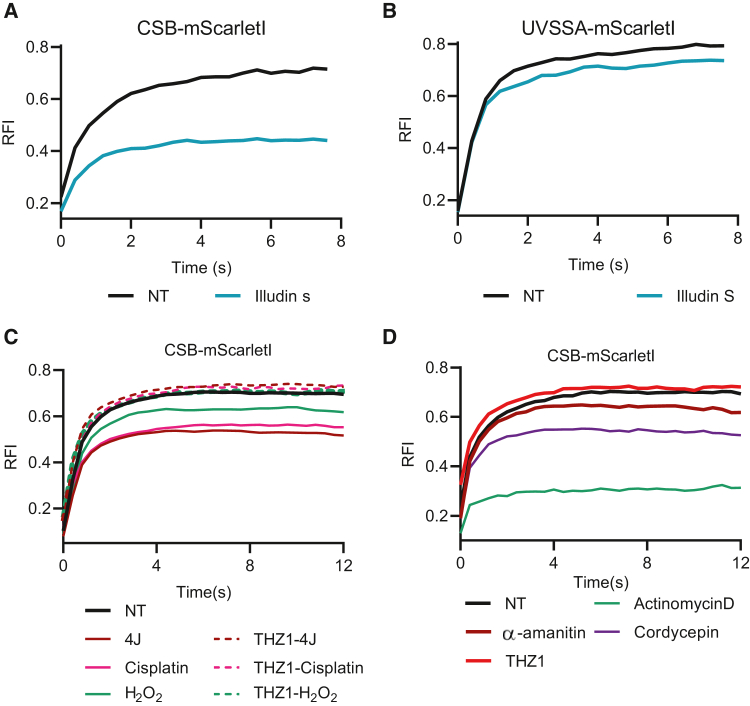
CSB immobilization as general transcription stress marker (A and B) FRAP analysis of mScarletI-tagged CSB (A) and UVSSA (B) in NT cells and after 2 h treatment with 30 ng/mL Illudin S. Illudin S is a natural compound from mushroom *O. illudins*, causing DNA lesions that are repaired by TC-NER. Plotted curves represent the average of 2 independent experiments of >17 cells in total. (C) FRAP analysis of mScarletI-tagged CSB in NT cells or after 1 h of treatment with the indicated DNA damage reagents including UV-C (4 J/m^2^), H_2_O_2_ (150 μM), and cisplatin (200 μM) and, where indicated, pre-treated with the transcription inhibitor THZ1 (1 μM) for 90 min. Plotted curves represent the average of 2 independent experiments of 10 cells per experiment and condition. (D) FRAP analysis of mScarletI-tagged CSB in NT cells and after 1 h treatment of THZ1 (1 μM), Cordycepin (10 μM), actinomycin D (1 μg/mL), and α-amanitin (100 μg/mL). THZ1 inhibits the phosphorylation of Ser5 of the RPB1 C-terminal domain (CTD) by the Cdk7 kinase. Cordycepin is a 3′ deoxy adenosine analog that stalls chain elongation when incorporated into the mRNA. Actinomycin D is a DNA intercalator. α-Amanitin traps RNA Pol II in a conformation that prevents translocation of the transcript and thereby inhibits nucleotide incorporation. Plotted curves represent the average of 2 independent experiments of >10 cells per experiment per condition.

The time-resolved immobilization upon DNA damage of especially mScarletI-tagged CSB shows that FRAP of TC-NER factors is a sensitive live-cell approach to detect UV-induced transcription-blocking DNA damage and its repair. To study whether TC-NER factor immobilization is also observed upon exposure to different types of TBLs, we tested the effect of Illudin S, which generates DNA lesions that are poor substrates for GG-NER but efficiently induces TC-NER.^53^ Similar to UV treatment, mScarletI-tagged CSB was strongly immobilized upon Illudin S treatment (Figure 4A), while the immobilization of UVSSA was more modest (Figure 4B). This difference between CSB and UVSSA immobilization was also observed upon UV-induced damage and confirms that mScarletI-tagged CSB is a more sensitive TC-NER factor to study TC repair and TBLs.

To test whether CSB FRAP can be used for a wide spectrum of structural diverse TBLs, we tested different types of DNA-damaging agents that have been described to impede transcription. The platinum drug cisplatin has been reported to induce DNA inter-strand and intra-strand crosslinks, which block transcription and result in TC-NER initiation.^6^^,^^54^^,^^55^^,^^56^^,^^57^^,^^58^ In line, cisplatin exposure strongly immobilized mScarletI-tagged CSB (Figure 4C) to a similar extent as UV treatment at 4 J/m^2^. Similar to upon UV, cisplatin-induced CSB immobilization was fully dependent on active transcription. CSB is also immobilized upon induction of oxidative lesions generated by H_2_O_2_ in a transcription-dependent way (Figure 4C). It has been suggested that CSB has a role in pushing RNA Pol II over less bulky DNA lesions,^59^^,^^60^ such as those generated by oxidative stress. This may result in a rather modest CSB immobilization, as the binding time of CSB would be more transient, which could explain the relatively small immobilization of CSB even under the exposure at high dose of H_2_O_2_. Alternatively, considering the massive amount of DNA lesions generated by this high concentration of H_2_O_2_ treatment in CSB FRAP, it is possible that base excision repair intermediates, rather than the oxidative damage itself, block elongating RNA Pol II, which might induce TC-NER or cause additional CSB stalling.^43^

### DNA-damage-independent impediment of RNA Pol II elongation immobilizes CSB

In addition to DNA damage, we also tested whether other impediments of RNA Pol II forward translocation would induce CSB immobilization. To test this, we exposed cells to four different transcription inhibitors that function in different stages of the transcription cycle (Figure 4D). First, we performed FRAP on mScarletI-tagged CSB cells after treatment for 1 h with 1 μg/mL actinomycin D, a DNA intercalator^61^ known to inhibit transcription elongation completely.^39^^,^^62^^,^^63^ Actinomycin D almost completely traps elongating RNA Pol II at the DNA, as observed in GFP-tagged RNA Pol II live-cell imaging studies.^39^^,^^62^ In line with such RNA Pol II trapping, actinomycin D treatment led to a very severe reduced mScarletI-tagged CSB mobility (Figure 4D). Cordycepin, a chain-terminating nucleoside analog,^64^ also impedes forward translocation of elongating RNA Pol II but to a much lower extent than actinomycin D.^39^ This explains the fact that Cordycepin treatment immobilized CSB, albeit to a lower extent than actinomycin D (Figure 4D). Interestingly, α-amanitin, which traps RNA Pol II in a conformation to prevent nucleotide incorporation resulting in a severe reduction of the elongation rate,^39^ did not lead to a severe increase in CSB immobilization (Figure 4D). The relatively minor CSB immobilization after exposure of this RNA Pol II-stalling drug can be explained by the fact that RNA Pol II is swiftly degraded by α-amanitin,^39^^,^^63^ thereby most likely leading to CSB release from the chromatin.

In contrast to the above-mentioned inhibitors that impede the forward progression of elongating RNA Pol II, inhibition of transcription initiation by the CDK7 inhibitor THZ1^47^^,^^65^^,^^66^ results in a minimal mobilization of CSB (Figure 4D). This chromatin release of CSB upon THZ1 treatment can most likely be explained by the transient binding of CSB to elongating RNA Pol II. As THZ1 will strongly reduce the quantity of elongating RNA Pol II,^39^^,^^63^ the transient binding of CSB to elongating RNA Pol II will also be severely reduced, resulting in the observed subtle CSB mobilization. Together, the changes in chromatin binding of mScarletI-tagged CSB, as determined by FRAP, in response to transcription inhibitors further support the close relationship between CSB chromatin binding and impeded forward translocation of elongating RNA Pol II and indicate that our developed mScarletI-tagged CSB KI cell line is a highly sensitive live-cell marker to study RNA Pol II elongation interference.

### CSB-mScarletI fluorescence to quantify CSB protein levels

**Figure 5:**
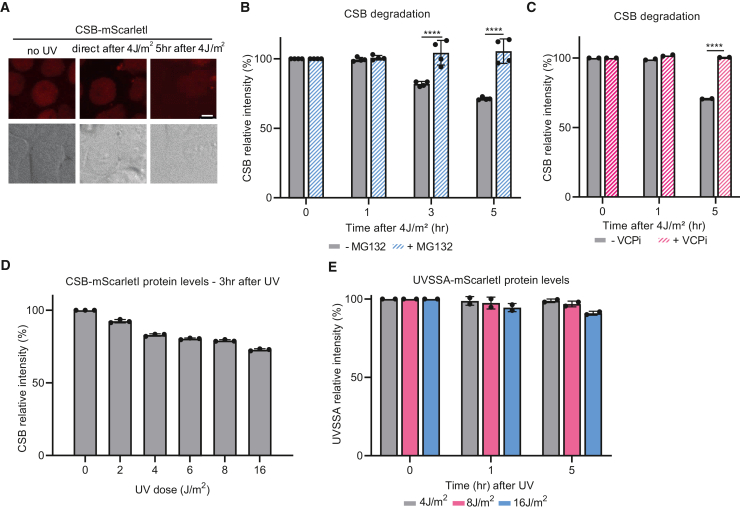
UV-induced CSB degradation determined by flow cytometry (A) Representative images of mScarletI-tagged CSB at indicated time points after UV-induced DNA damage (4J/m^2^). Scale bar: 5 μm. (B and C) Mean ± SD of mScarletI-tagged CSB FI as a measure for CSB protein levels by flow cytometry after UV irradiation (4 J/m^2^) with or without pre-treatment for 1 h with (B) proteasome inhibitor (MG132, 50 μM) or (C) VCP inhibitor (VCPi; 5 μM). n = 4 (MG132), n = 2 (VCPi), >5,000 cells were collected in each individual experiment. ∗∗∗∗p ≤ 0.0001 analyzed by two-way ANOVA. (D and E) Graphs indicate average FI of mScarletI-tagged CSB or UVSSA cells upon exposure to the indicated UV dose, analyzed by flow cytometry as a measure for CSB and UVSSA protein levels upon indicated treatments. n = 3 (CSB), n = 2 (UVSSA) mean ± SD, >5,000 cells were collected in each individual experiment.

Previously, CSB was shown to be targeted for p97/VCP and proteasome-mediated degradation following DNA damage.^15^^,^^21^^,^^22^ In line with these findings, we also observed a UV-induced decrease of CSB protein levels as determined by mScarletI fluorescence in live-cell imaging experiments (Figure 5A). To efficiently quantify CSB degradation upon UV damage, we determined mScarletI-tagged CSB fluorescence levels, a direct measurement for CSB protein abundance, by flow cytometry (Figure 5B). mScarletI-tagged CSB fluorescence remained unchanged within the first hour after UV irradiation, suggesting that lesion-stalled CSB is not directly degraded, while at this time point, it is strongly immobilized (Figure 3C). However, total CSB protein levels were significantly reduced ranging from ∼20% (3 h) to ∼30% (5 h) after UV irradiation at 4 J/m^2^, suggesting that CSB is degraded during the cellular response to transcription-blocking DNA damage.

To confirm whether the UV-induced loss of CSB is caused by proteasome degradation, we determined CSB protein levels (Figure 5B) in the presence of the proteasome inhibitor MG132. Proteasome inhibition fully blocked the UV-induced reduction of CSB protein levels. Furthermore, in line with previous findings,^55^ the proteasomal degradation of CSB was dependent on p97/VCP, which segregates ubiquitylated proteins from chromatin or protein complexes before proteasomal degradation^67^ (Figure 5C). As CSB is degraded over time, this could indicate that CSB is degraded during TC-NER. If this were the case, CSB degradation would be expected to be dose dependent. In line with this hypothesis, the reduction of mScarletI-tagged CSB fluorescence showed a distinct UV-dose dependency 3 h after UV irradiation, ranging from ∼10% at 2 J/m^2^ to ∼30% at 16 J/m^2^ (Figure 5D). Degradation of TC-NER factors is not generally observed and seems specific for CSB, as mScarletI-tagged UVSSA protein levels quantified by flow cytometry were hardly affected upon UV induction (Figure 5E).

### Downstream TC-NER factors affect CSB protein levels and chromatin binding

**Figure 6:**
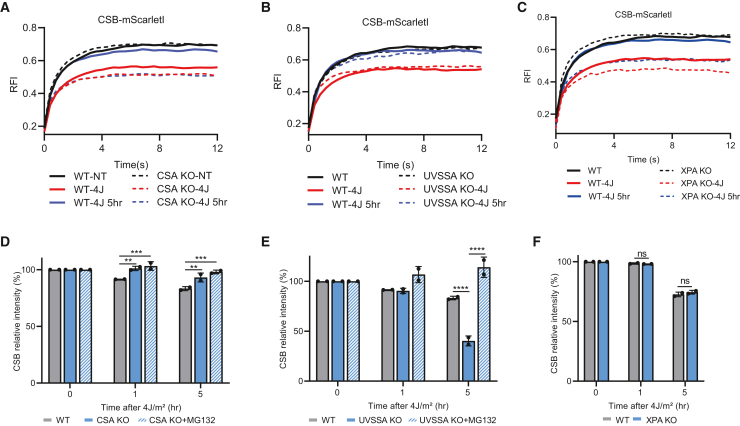
TC-NER activity determined by CSB immobilization (A–C) FRAP analysis of mScarletI-tagged CSB KI in WT, TC-NER-deficient CSA, UVSSA, and XPA KO cells in non-perturbed condition (NT) immediately after UV irradiation (4 J/m^2^) or 5 h after UV irradiation (4 J/m^2^). Plotted curves represent the average of 3 (A and C) or 2 (B) independent experiments of 10 cells per experiment and condition. (D–F) FI of mScarletI-tagged CSB KI in WT or TC-NER-deficient CSA, UVSSA, and XPA KO cells at the indicated treatment conditions were analyzed by flow cytometry. Where indicated, cells were pre-treated for 1 h with proteasome inhibitor (MG132, 50 μM). n = 2 mean ± SD, >5,000 cells were collected in each individual experiment. ∗∗p ≤ 0.01, ∗∗∗p ≤ 0.001, ∗∗∗∗p ≤ 0.0001, analyzed by two-way ANOVA.

Next, we set out to test the effects of downstream TC-NER factors on CSB chromatin binding and protein levels. Therefore, we generated CSA, UVSSA, and XPA KO cells using CRISPR-Cas9-mediated genome editing in mScarletI-tagged CSB KI cells. CSA, UVSSA, and XPA KO clones were confirmed by genotyping or western blot analysis (Figure S1C). All TC-NER KO cells were hypersensitive to UV-induced DNA damage (Figure S1D) and were deficient in transcription recovery (Figure S1E), confirming that these KO cells are fully TC-NER deficient. In the absence of CSA, UVSSA, and XPA, CSB was still immobilized directly after UV irradiation to a comparable level as that observed in TC-NER-proficient cells (Figures 6A–6C). This observation indicates that TBL-induced CSB immobilization is independent of CSA, UVSSA, and XPA and indicates that these factors act downstream of CSB during TC-NER, in line with previous studies.^18^^,^^24^ Interestingly, the immobilization of CSB was slightly increased compared to WT cells and remained immobilized for 5 h in CSA KO and XPA KO cells (Figure 6A and 6C). This indicates that CSB remains bound at damaged chromatin in CSA- and XPA-deficient cells. This is most likely explained by the fact that due to TC-NER deficiency, TBLs cannot be repaired, and lesion-stalled RNA Pol II will accumulate over time, explaining the slightly bigger and prolonged immobile fraction of CSB present in CSA and XPA KO cells. Surprisingly, although UVSSA is, like CSA and XPA, an essential TC-NER factor, and therefore the TBLs cannot be removed by TC-NER, the mobility of mScarletI-tagged CSB was restored 5 h after UV irradiation in UVSSA KO cells (Figure 6B).

To investigate this unexpected observation in UVSSA KO cells, we studied CSB protein levels in these different TC-NER KO cells, as UVSSA, through its interaction with the deubiquitylating enzyme USP7, stabilizes CSB.^20^^,^^21^ In TC-NER-proficient cells, CSB-mScarletI was degraded, as shown by reduced protein levels after UV irradiation at 4 J/m^2^ as determined by flow cytometry (Figure 5). However, CSB protein levels remained largely unaffected in CSA KO cells upon DNA damage induction (Figure 6D). This supports the hypothesis that the CRL4^CSA^ E3 ligase complex targets CSB for proteasomal degradation.^15^

In contrast to CSA KO cells, loss of UVSSA triggers massive loss of CSB 5 h after UV irradiation, which could be fully rescued by proteasome inhibition (Figure 6E). This is in line with previous studies that demonstrated that UVSSA, via its interaction partner USP7, counteracts the degradation of CSB.^20^^,^^21^ This degradation of most likely chromatin-bound CSB in UVSSA KO cells can explain its mobilization 5 h after UV, as in this scenario, the residual CSB proteins represent a free, non-chromatin-bound fraction. In XPA KO cells, no difference in CSB levels compared to TC-NER-proficient WT cells was observed, indicating that the regulation of CSB degradation upon DNA damage happens in reaction steps upstream of XPA. Importantly, these FRAP data in TC-NER-deficient cells show that our developed CSB-mScarletI KI cells are a sensitive tool to study TC-NER progression by FRAP and, furthermore, allow us to study CSB degradation by easy quantification by flow cytometry.

### CSB mobility and stability are regulated by the CRL4^CSA^ complex and USP7

**Figure 7:**
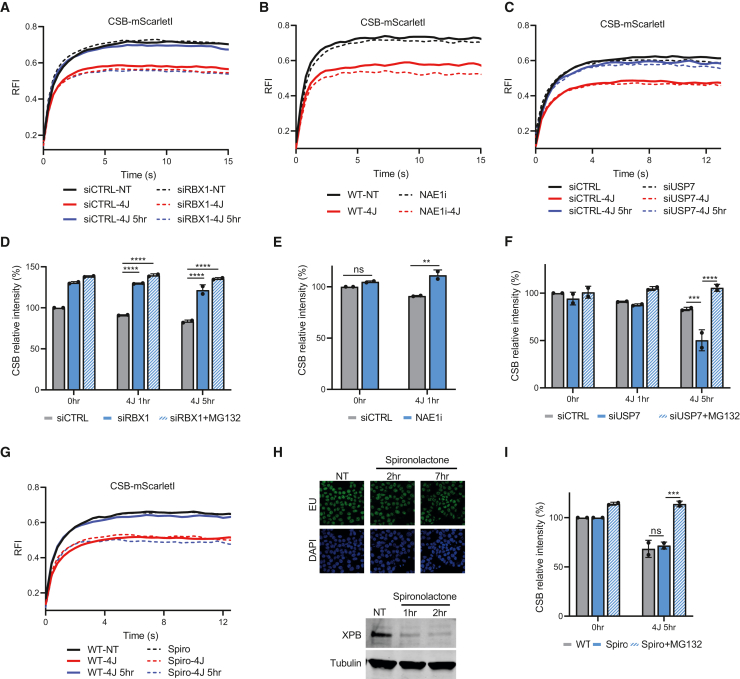
CSB immobilization and protein levels are regulated by CRL4^CSA^ and USP7 (A and C) FRAP analysis of mScarletI-tagged CSB KI after control siRNA transfection (siCTRL) or siRNA-mediated gene knockdown of RBX1 or USP7 as indicated in non-perturbed conditions (NT) immediately after irradiation (4 J/m^2^) or 5 h after irradiation (4 J/m^2^). Plotted curves represent the average of 2 independent experiments of 10 cells per experiment and condition. (B) FRAP analysis of mScarletI-tagged CSB KI with or without NAE1i treatment in non-perturbed conditions (NT) or immediately after irradiation (4 J/m^2^). Plotted curves represent the average of 2 independent experiments of 10 cells per experiment and condition. (D–F) FI of mScarletI-tagged CSB KI cells representing CSB protein levels at the indicated treatment conditions were analyzed by flow cytometry. mScarletI-tagged CSB KI cells were transfected with control siRNA or indicated siRNAs targeted at RBX1 or USP7 and pre-treated with MG132 (50 μM) as indicated (D and F). NAE1i was added to mScarletI-tagged CSB KI cells 1 h before UV irradiation (E). Time points indicate how long cells were left to recover before beginning flow cytometry measurements. n = 2 mean ± SD, >5,000 cells were collected in each individual experiment. ∗∗p ≤ 0.01, ∗∗∗p ≤ 0.001, ∗∗∗∗p ≤ 0.0001, analyzed by two-way ANOVA. (G) FRAP analysis of mScarletI-tagged CSB KI in non-perturbed conditions (NT) immediately after irradiation (4 J/m^2^) or 5 h after irradiation (4 J/m^2^) with or without 2 h pre-treatment with spironolactone (10 μM). Plotted curves represent the average of 2 independent experiments of 10 cells per experiment and condition. (H) Representative images (top) of mScarletI-tagged CSB KI cells that were pulse labeled with EU for 1 h followed by Click-iT-chemistry-based EU coupling to Alexa Flour (488 nm) of cells NT and after spironolactone treatment (10 μM) for 2 or 7 h. Bottom: western blot analysis of XPB of whole-cell lysates of mScarletI-tagged CSB KI cells NT or treated for the indicated times with spironolactone (10 μM). Tubulin is used as loading control. (I) FI of mScarletI-tagged CSB KI cells representing CSB protein levels 5 h after UV, with or without spironolactone treatment (10 μM) or MG132 (50 μM), was analyzed by flow cytometry. n = 2 mean ± SD, >5,000 cells were collected in each individual experiment. ∗∗∗p ≤ 0.001 analyzed by two-way ANOVA.

To test whether the loss of CSB remobilization and degradation in CSA KO cells is indeed caused by the CRL4^CSA^ E3 ligase activity^14^ and not by, e.g., disrupted conformation of the TC-NER complex or absence of CSA-binding partners, we studied CSB stability and protein levels upon small interfering RNA (siRNA)-mediated depletion of Rbx1, the essential component of SCF E3 ubiquitin ligases.^13^ Depletion of Rbx1 resulted in stronger and prolonged immobilization of CSB (Figure 7A) to an extent similar to that in CSA KO (Figure 6A). Additionally, CSB protein levels did not show a UV-induced degradation (Figure 7D), suggesting that the CRL4^CSA^ E3 ligase complex is responsible for the proteasomal degradation of CSB. Similar data were obtained by inhibiting the neddylation of the CRL4^CSA^ E3 ligase complex by a NAE1 inhibitor (MLN4924) (Figure 7B and 7E), which is crucial for the activation of the ubiquitin activity of CRL4^CSA^. Taken together, these data indicate that the CRL4^CSA^ E3 ligase complex is crucial for the proteasomal degradation of CSB.

Similarly, we tested whether the CSB degradation observed in UVSSA KO cells (Figure 6B) was due to the loss of USP7 targeting to the TC-NER complex or was, for example, caused by the loss of TFIIH recruitment in UVSSA KO cells.^23^^,^^24^ The observed CSB degradation in UVSSA KO cells is most likely caused by the absence of USP7 in the TC-NER complex, as siRNA-mediated USP7 depletion increased the UV-induced degradation of CSB to a similar extent as that observed in UVSSA KO cells (Figure 7F). Similar to in UVSSA KO cells, in USP7-depleted cells, CSB was remobilized 5 h after UV damage, indicating that normal degradation of CSB is a mechanism that reduces CSB chromatin binding (Figure 7C). To exclude the role of TFIIH, we inactivated TFIIH by chemical depletion of the crucial ATP-dependent helicase subunit XPB by spironolactone,^68^^,^^69^ which, at these time points, did not influence transcription levels (Figure 7H). In contrast to USP7 depletion, XPB degradation resulted in a prolonged CSB immobilization (Figure 7G) without affecting CSB levels (Figure 7I). This indicates that the effects observed in UVSSA KO cells are caused by the loss of USP7-mediated deubiquitylation and not by the loss of TFIIH recruitment. Together, these data show that the E3 ligase activity of CRL4^CSA^ and the deubiquitylation activity of USP7 are key factors for the regulation of CSB levels during TC-NER and thereby play an important role in the binding and release of CSB from damaged chromatin. Also, TFIIH and XPA are important for the remobilization of CSB but most likely not by regulation of CSB degradation, which happens upstream of TFIIH and XPA.

## Discussion

DNA damage that blocks transcription severely impacts cell function^7^; however, thus far, no sensitive live-cell markers have been present to detect and quantify transcription-blocking DNA damage. Many live-cell assays that study DNA damage induction and repair make use of the fact that DNA repair proteins, which normally freely diffuse in the nucleus, bind to chromatin upon DNA damage. A powerful tool to study DNA damage-induced chromatin binding in living cells is FRAP^38^^,^^48^; however, for exogenously expressed TC-NER proteins, this assay was not very sensitive,^21^^,^^70^ most likely due to their high expression levels. In this study, we made use of CRISPR-Cas9-mediated genome editing to fluorescently label endogenous CSB and UVSSA, which allowed us for the first time to study dynamic chromatin binding and protein levels of these endogenously expressed TC-NER proteins in living cells.

Endogenous fluorescent-tagged CSB expression resulted in a striking increase in sensitivity in chromatin binding as assessed by FRAP for CSB, clearly indicating the importance of analyzing proteins expressed at endogenous levels. FRAP allowed us to detect CSB chromatin binding at low and physiologically relevant damage loads (e.g., 2 J/m^2^ UV) and showed that almost 50% of all CSB is bound at 8 J/m^2^. Interestingly, while both CSB and UVSSA are essential for TC-NER, and both are expected to participate in TC-NER with equal stoichiometry,^5^ CSB-mScarletI immobilization was much more pronounced than UVSSA-mScarletI upon induction of TBLs. This might be partially caused by the fact that UVSSA is expressed approximately 2-fold higher than CSB, resulting in more non-chromatin-bound UVSSA compared to CSB and therefore a relative smaller fraction of immobilized UVSSA. However this 2-fold difference in expression cannot explain the big difference in CSB and UVSSA immobilization observed: respectively, 50% versus 13% immobile fraction at 8 J/m^2^. Furthermore, CSB and UVSSA accumulation kinetics (mainly k_on_) at sites of local damage were very similar. Therefore, the difference in immobilization most likely reflects a difference in residence time (k_off_) of UVSSA and CSB at lesion-stalled RNA Pol II. This suggests that CSB might have additional activities compared to UVSSA, explaining its longer residence time. In line with such a hypothesis, CSB binding to lesion-stalled RNA Pol II is needed for stable UVSSA incorporation in the TC-NER complex^21^^,^^24^; this could explain the longer residence time of CSB compared to UVSSA. Furthermore, in contrast to UVSSA, CSB plays an important role to discriminate lesion-stalled RNA Pol II from other non-forward-translocating RNA Pol II, e.g., RNA Pol II at natural pausing sites,^5^^,^^11^^,^^12^ by promoting the forward movement of RNA Pol II. In line, CSB immobilization was not only observed upon induction of TBL (e.g., UV or IlludinS) but also upon inhibiting the forward translocation of RNA Pol II by transcription inhibitors like Cordycepin or α-amanitin.^63^ Together, these results show that CSB FRAP is a highly sensitive tool to study general transcription impediments, for example, induced by DNA damage or other forms of transcription impediments.

Importantly, the damage-load-dependent fraction of chromatin-bound CSB proteins provides us with a large dynamic window to study CSB chromatin binding and therefore allowed us to precisely follow CSB chromatin binding in time. 5 h after UV-induced DNA damage, almost all CSB is remobilized. This remobilization reflects repair efficiency, as this remobilization is not observed in TC-NER-deficient CSA and XPA KO cells or upon chemical XPB depletion. Therefore, the time-resolved CSB remobilization can be used as a single-cell readout for TC-NER efficiency and allows us to detect perturbations in repair. Furthermore, the use of these TC-NER-deficient cells allows us to study the sequential TC-NER complex assembly upon TBL induction.

Apart from monitoring changes in chromatin binding of CSB and UVSSA by live-cell imaging, flow cytometry analysis allowed us to efficiently quantify CSB and UVSSA protein abundance upon DNA damage. In contrast to UVSSA, CSB is degraded, especially at later time points upon DNA damage, in a proteasome- and p97/VPC-dependent manner. Thus far, the exact role of CSB degradation has not been well understood. However, it is tempting to speculate that CSB might be degraded during the TC-NER reaction. CSB pushes RNA Pol II forward to identify lesion-stalled RNA Pol II.^5^^,^^11^^,^^34^ Therefore, the CSB activity to push RNA Pol II forward might form a barrier for repair, as most likely, CSB will block RNA Pol II backtracking. RNA Pol II backtracking is important to provide repair proteins access to the TBL, which is otherwise shielded by RNA Pol II. Therefore, we hypothesize that CSB needs to be degraded to allow RNA Pol II backtracking. As shown by our data, and in line with previous studies,^67^^,^^71^^,^^72^^,^^73^^,^^74^^,^^75^^,^^76^ CSB is ubiquitylated after UV irradiation, subsequently making it a substrate for VCP. Subsequently, VCP-extracted ubiquitylated CSB is targeted for proteasomal degradation. Unlike CSB, UVSSA is hardly degraded upon DNA damage, indicating that UVSSA has no activities that might perturb the TC-NER reaction, and is, for example, only involved in the recruitment of downstream repair proteins like TFIIH.^18^^,^^21^^,^^23^^,^^24^^,^^52^

In line with the need of CSB degradation for its release during TC-NER, we observed an increased UV-induced CSB immobilization in cells in which the CRL4^CSA^ activity is inhibited (e.g., RBX1 knockdown, CSA KO, or NEDD8-activating enzyme inhibitor [NAEi]), and thus CSB degradation is strongly reduced.^14^ This increase in immobilization upon CRL4^CSA^ inhibition is more severe compared to what has been observed in XPA KO cells or upon XPB degradation, indicating that indeed, perturbed CSB degradation results in prolonged CSB binding at TBLs after UV damage. Vice versa in conditions where CSB is no longer stabilized due to the absence of USP7 deubiquitylating activity, CSB degradation is strongly increased, which correlates with CSB release from the chromatin 5 h after UV. These data indicate that, most likely, chromatin-bound CSB is degraded, while freely diffusing CSB in the nucleus is not targeted for degradation. Whether CSB needs to be degraded during the TC-NER reaction in WT cells remains unclear. However, in WT cells, CSB degradation is observed at time points when CSB is remobilized, suggesting that at least a part of the observed CSB remobilization is caused by TBL-bound CSB degradation by CRL4^CSA^.

This study shows that the mScarletI-tagged UVSSA and CSB KI cells provide a powerful tool to study endogenous TC-NER factors in living cells. This approach allows us to detect TBLs at physiologically relevant doses as well as their repair in a sensitive manner by FRAP. Additionally, the use of these cell lines will also allow us to study TC-NER complex assembly and stability in the biologically relevant setting of the living cell.

### Limitations of the study

While this study shows the sensitivity and wide applicability of the use FRAP in combination with endogenous-tagged proteins involved in chromatin-related processes, the generation of homozygous fluorescent-tagged KI cell lines may be laborious and time consuming. Furthermore, tagging proteins with a relatively big fluorescent protein may interfere with its function. Therefore, it is crucial to carefully confirm the correct cellular functionality of the tagged proteins before performing live-cell imaging experiments. Finally, live-cell imaging using endogenously expressed proteins may be difficult for low-expressed proteins, as their detection might only be possible with high laser intensities, resulting in monitor bleaching, which will impede their applicability.

## STAR★Methods

### Key resources table

**Table:** 

REAGENT or RESOURCE	SOURCE	IDENTIFIER
**Antibodies**		

CSB/ERCC6 (1:1500)	SantaCruz	Cat # sc10459; RRID:AB_668957
HA (3F10) (1:1000)	Roche	Cat # sc10459; RRID:AB_668957
CSA/ERCC8 (1:1500)	Abcam	Cat# ab137033; RRID:AB_2783825
USP7 (1:200)	Bethyl	Cat# A300-033A; RRID:AB_203276
XPA (1:2000)	Genetex	Cat# GTX103168; RRID:AB_10730673
XPB (1:1500)	Abcam	Cat# ab190698; RRID:AB_3076479
RNA Pol II Ser2-P (1:1000)	Chromotek	Cat# 3e10; RRID:AB_2631403
p62/GTF2H1 (1:1500)	Sigma	Cat# WH0002965M1; RRID:AB_1843930
Tubulin (1:5000)	Sigma	Cat# T5168; RRID:AB_477579

**Oligonucleotides**		

Genotyping primers:		
CSB KI-FW1	CACCTGCAGGAAGCTTCTGC	CSB KI-front
CSB KI-RV1	CAATCCAAGTATTTTCTCCTTTAGC	CSB KI-front
CSB KI-FW2	CACCACAGAACACGATGACC	CSB KI-front
CSB KI-RV2	TCCATGTGCACCTTGAACCG	CSB KI-front
CSB KI-FW3	CATCCGGAGCTTGCAGGATCG	CSB KI-back
CSB KI-RV3	TCTCCTTTAGCTAGCATTATTA	CSB KI-back
UVSSA KI-FW1	ACGCGGATTTCGGCTCCAAC	UVSSA KI-back
UVSSA KI-RV1	TTCTGCGAGGCCAGACCCAT	UVSSA KI-back
UVSSA KI-FW2	ATCCTGCTCCCCGGAATGCC	UVSSA KI-front
UVSSA KI-RV2	CCACCGCTTGATTTTTGGCAGG	UVSSA KI-front
UVSSA KO-FW	GTAAAGGCCTTGCTGGACAC	UVSSA KO-TIDE
UVSSA KO-RV	GAAGAGAAGCACCAACCACAG	UVSSA KO-TIDE

**Other**		

siRNA sequences		
non targeting siRNA#5	D-001210-05-20	UGGUUUACAUGUCGACUAA
siCSB	J-004888-09	GCAUGUGUCUUACGAGAUA
siUSP7	LQ-006097-00-0005	AAGCGUCCCUUUAGCAUUA, GCAUAGUGAUAAACCUGUA, UAAGGACCCUGCAAAUUAU, GUAAAGAAGUAGACUAUCG
siCSA	L-011008-00-0005	GUAAAGCAGUGUGUUCCAU, CAGACAAUCUUAUUACACA, CAUCAUAUGUCUCCAGUCU, GAUUGUACUUUAUGACCUU
siRBX1	L-004087-00-0005	GAAGCGCUUUGAAGUGAAA, GGGAUAUUGUGGUUGAUAA, GGAACCACAUUAUGGAUCU, CAUAGAAUGUCAAGCUAAC
sgRNA sequences:		
sgCSB	AATGTTGTTTAGCAGTATTC	CSB KI
sgUVSSA-1	CTACGCACTGAACTAGAGAG	UVSSA KI
sgCSA	GTCCGCACGCCAAACGGGTT	CSA KO
sgUVSSA-2	GAGACGGTTGTAAATGAGCA	UVSSA KO
sgXPA	GTATCGAGCGGAAGCGGCAG	XPA KO

### Resource availability

#### Lead contact

Further information and requests for resources and reagents should be directed to and will be fulfilled by the lead contact, Jurgen Marteijn (J.Marteijn@erasmusmc.nl).

#### Materials availability

Plasmids and cell lines generated in this study will be made available upon reasonable request.

#### Data and code availability

• All data reported in this paper, including additional raw imaging data, is available from the lead contact upon request.
• This study does not report original code.
• Any additional information required to reanalyze the data reported in this paper is available from the lead contact upon request.

### Experimental model and study participant details

#### Cell culture

HCT116 colorectal cancer cells and RPE retinal pigment epithelium cells were cultured in a 1:1 mixture of DMEM (Gibco) and Ham’s F10 (Invitrogen) supplemented with 10% fetal calf serum (FCS, Biowest) and 1% penicillin-streptomycin in a humidified incubator at 37°C and 5% CO_2_. TC-NER factor knock-in (KI) cells were generated in HCT116 osTIR1 cells^42^ or RPE cells by transiently transfecting cells with a sgRNA-containing pLentiCRISPR.v2 plasmid (sgRNA sequences in Key Resources Table) targeting the stop codon of CSB or UVSSA and co-transfecting a homology-directed repair template, which included an Auxin-inducible Degron, fluorescent mScarletI-tag, HA-tag, hygromycin resistance cassette^42^ and homology arms (200 bp for CSB and UVSSA). Subsequently, cells were seeded in a low density to allow expansion and were kept in the presence of 100 μg/mL Hygromycin for two weeks to select for successful recombination. Single-cell clones were genotyped and homozygous KI clones were selected for further analysis. HCT116 knock-out cells were generated by transiently transfecting HCT116 osTIR1 CSB KI cells with a pLentiCRISPR.v2 plasmid containing appropriate sgRNAs (Key Resources Table). Transfected cells were selected using 1 μg/mL Blasticidin (Invitrogen) for 7 days and single cells were seeded to allow expansion. Genotyping of single-cell KO clones was performed by genomic PCR (primers in Key Resources Table) or KO was confirmed by immunoblotting (antibodies in Key Resources Table).

### Method details

#### RNA interference

siRNA transfection was performed using Lipofectamine RNAiMAX (Invitrogen) transfection reagent, according to the manufacturer’s instructions. The siRNA oligonucleotides used (Dharmacon) are listed in Key Resources Table.

#### Clonogenic survival assay

Cells were seeded in triplicate in 6-well plates (300 cells/well) and were the following day treated with the indicated DNA damaging agents. After 1 week, colonies were fixed and stained in 50% methanol, 7% acetic acid, and 0.1% Coomassie blue and subsequently counted with the Gelcount (Oxford Optronix, Software Version 1.1.2.0). The survival of at least 2 independent experiments was plotted as the mean percentage of colonies detected following the indicated treatment dose, compared to the mean number of colonies from the non-treated samples which was set at 100%.

#### Western blot and antibodies

Lysates were separated by sodium dodecyl sulphate-polyacrylamide gel electrophoresis (SDS–PAGE) and transferred to a Polyvinylidene difluoride (PVDF) membrane (0.45 μm). Membranes were blocked with 5% BSA in PBS for 1 h at room temperature and incubated with primary antibodies (Key Resources Table). After washing of the blots in PBS tween, secondary antibodies (Key Resources Table) coupled to IRDyes (LI-COR) were used to visualize proteins using an Odyssey CLx infrared scanner (LiCor).

#### EU incorporation

Cells were seeded on coverslips at least 2 days prior to experiments. Seeded cells were pulse-labeled with 200 mM 5′ethynyl uridine (EU, Jena Bioscience) in culture medium for 30 min before fixation with 3.7% formaldehyde (FA, Sigma) at room temperature for 15 min. After permeabilization with 0.5% Triton-100 in PBS, Click-iT azide-based reaction was performed as described in the manufacturer’s manual. DAPI was added to visualize the nuclei. Images were captured using a Zeiss LSM 700 confocal and quantified by ImageJ as integrated intensity.

In order to measure the recovery of transcription rate after UV, cells were mock-treated or irradiated with 8J/m^2^ UV-C, 2 or 24 h before EU incorporation.

#### Live-cell confocal laser-scanning microscopy

For FRAP analysis, a Leica TCS SP8 microscope (LAS AF software, Leica) equipped with an HC PL APO CS2 63x 1.40 NA oil immersion lens (CSB, UVSSA) was used. Cells were maintained at 37°C and at 5% CO2 during imaging. FRAP was performed in the FRAP-wizard of the Lecia imaging software. A narrow strip of 512 × 16 lines (for CSB and UVSSA) spanning the nucleus was imaged every 400 ms for CSB and 200 ms for UVSSA during pre-bleach at an imaging speed of 400 Hz using a 561 nm laser. 5 frames (CSB, UVSSA) were measured to reach steady state fluorescence levels before photo-bleaching for 2 frames by 100% laser power for UVSSA, or 50% laser power for CSB. After photo-bleaching, the recovery of fluorescence was measured for 30 (CSB) or 20 (UVSSA) frames until steady-state fluorescence was reached. Fluorescence intensity was measured inside the nucleus and outside of the cell and recovery was determined by correcting for background signal (outside the cell) and normalizing the values to the average pre-bleach fluorescence intensities which were set at 1. To determine the immobile fraction (Fimm) from the FRAP measurements, we renormalized the data, using the fluorescence intensity recorded immediately after bleaching (I0) and the average fluorescence between 8 and 12s for CSB and between 5 and 8s for UVSSA after the start of the FRAP experiment (once recovery is complete) from the unchallenged cells (Ifinal, unc) and UV-irradiated cells (Ifinal, UV) and using the formula: Fimm = 1—(Ifinal, UV—I0, UV)/(Ifinal, unc—I0, UV).^48^

A Leica SP8 confocal microscope equipped with a 40× quartz objective was used for local UV-damage induction. Local DNA damage infliction for accumulation studies of mScarletI-tagged protein was performed using a 266 nm UV-C diode pumped solid-state laser (Rapp OptoElectronic, Hamburg) as described previously^21^^,^^45^ with some adaptions. Cells were grown on quartz cover-slips and were imaged and irradiated through a 40 ×1.2 numerical aperture (NA) Ultrafluar quartz objective. Damage was induced at 15% of maximum 266nm laser-power. Images were acquired using the LAS AF software (Leica) and the fluorescence intensity at the local damaged area was quantified over time, background corrected (fluorescence outside cells) and normalized to pre-damage fluorescence levels to determine accumulation kinetics.

#### Flow cytometry-based CSB and UVSSA protein quantification

Cells were seeded in 6-well plates at least 2 days prior to the treatment with indicated compounds or siRNAs. To assess the total CSB-mScarletI and UVSSA-mScarletI levels, adherent cells were trypsinized to acquire single-cell suspensions. Cells were pelleted by centrifugation for 5 min at 500 g, medium was aspirated and cell pellets were washed with 2% FCS in PBS for 2 times. After the final wash, cell pellets were resuspended in 500 μL PBS supplemented with 2% F BS and analyzed by flow cytometry. Flow cytometry analysis was performed using a BD LSRFortessa Cell Analyzer (BD Biosciences) equipped with a 561 nm laser and all flow cytometry data were analyzed with FlowJo software. At least 5,000 events were collected for each sample. Voltage settings for the FSC, SSC, and RFP channels were kept consistent for all experiments described. Single and viable cells were selected by gating using forward scatter (FSC-A) versus side scatter (SSC-A). The mScarletI fluorescence intensity was determined by creating a histogram plot for the red fluorescent protein (RFP) channel.

### Quantification and statistical analysis

Statistical analysis was performed using Prism9 (GraphPad Software). Details on how data and error bars are presented can be found in the figure legends. two-way ANOVA test was used to calculate significance between samples. p values expressed as ∗ p < 0.05; ∗∗ p < 0.01; ∗∗∗ p < 0.001 and ∗∗∗∗ p < 0.0001 were considered to be significant. ns, non-significant. Data presented in Figures 2C and 2D the area under the curve was analyzed by unpaired t-test. FRAP curves and calculated immobile fractions represents an average of the individual experiments, containing averaged data of 10 cells per experiment. All quantitative FRAP data from individual cells per experiment is depicted in Data S1.

## Supplemental information

Document S1. Figures S1–S4

Data S1. Single-cell fluorescence source data of FRAP experiments, related to Figures 2, 4, 6, and 7

Document S2. Article plus supplemental information
