# Supplementary material for: Live-cell imaging of endogenous CSB-mScarletI as a sensitive marker for DNA-damage-induced transcription stress
Source: Cell Rep Methods. 2024 Jan 3;4(1):100674. doi: 10.1016/j.crmeth.2023.100674 (PMC10831951; doi:10.1016/j.crmeth.2023.100674)
Supplement: Document S1. Figures S1–S4 [file mmc1.pdf]

Cell Reports Methods, Volume *4*

Supplemental information

Live-cell imaging of endogenous CSB-mScarletI as a sensitive marker for DNA-damage-induced transcription stress

Di Zhou, Qing Yu, Roel C. Janssens, and Jurgen A. Marteijn

Supplementary Figure 1

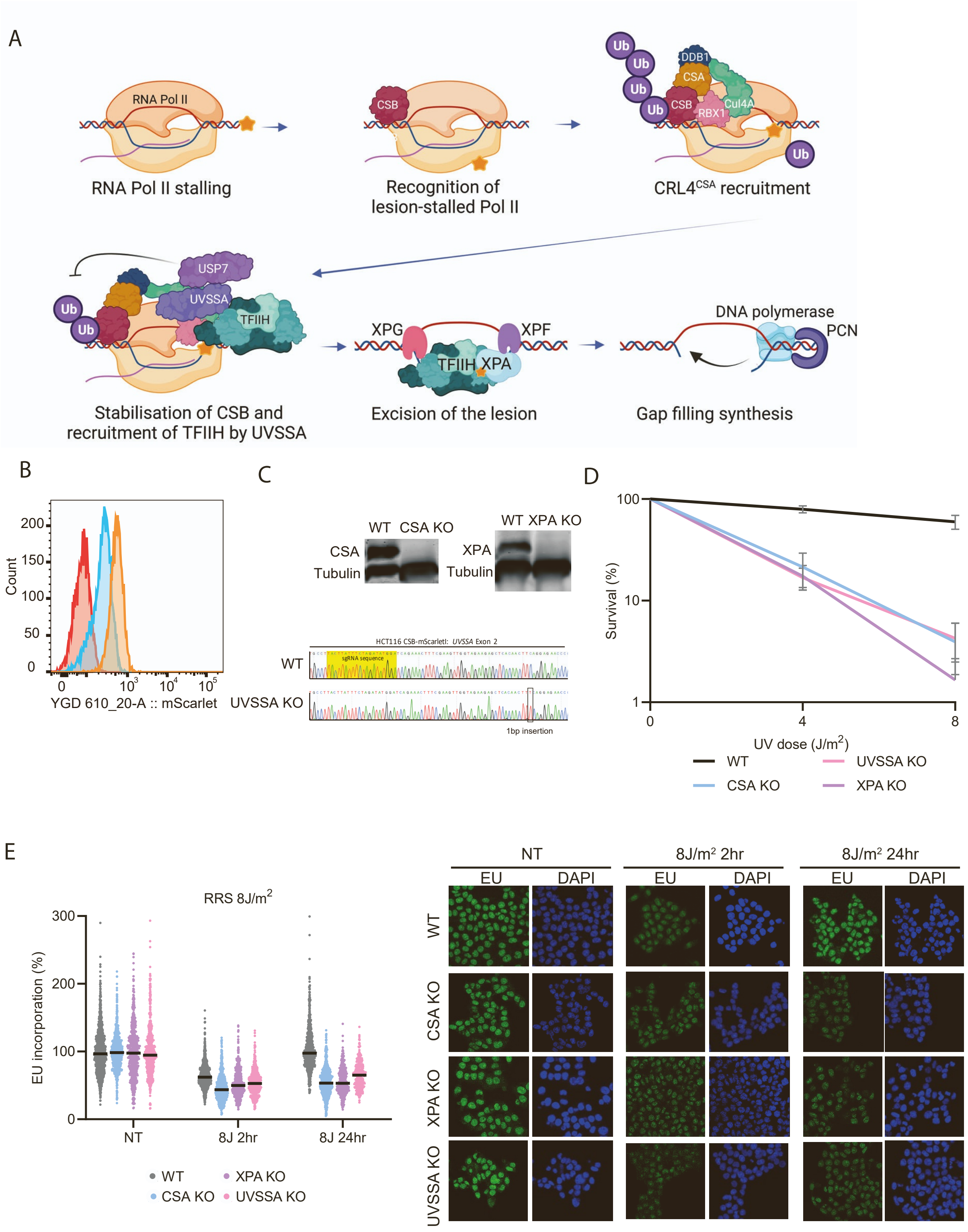

Supplemental Figure 2

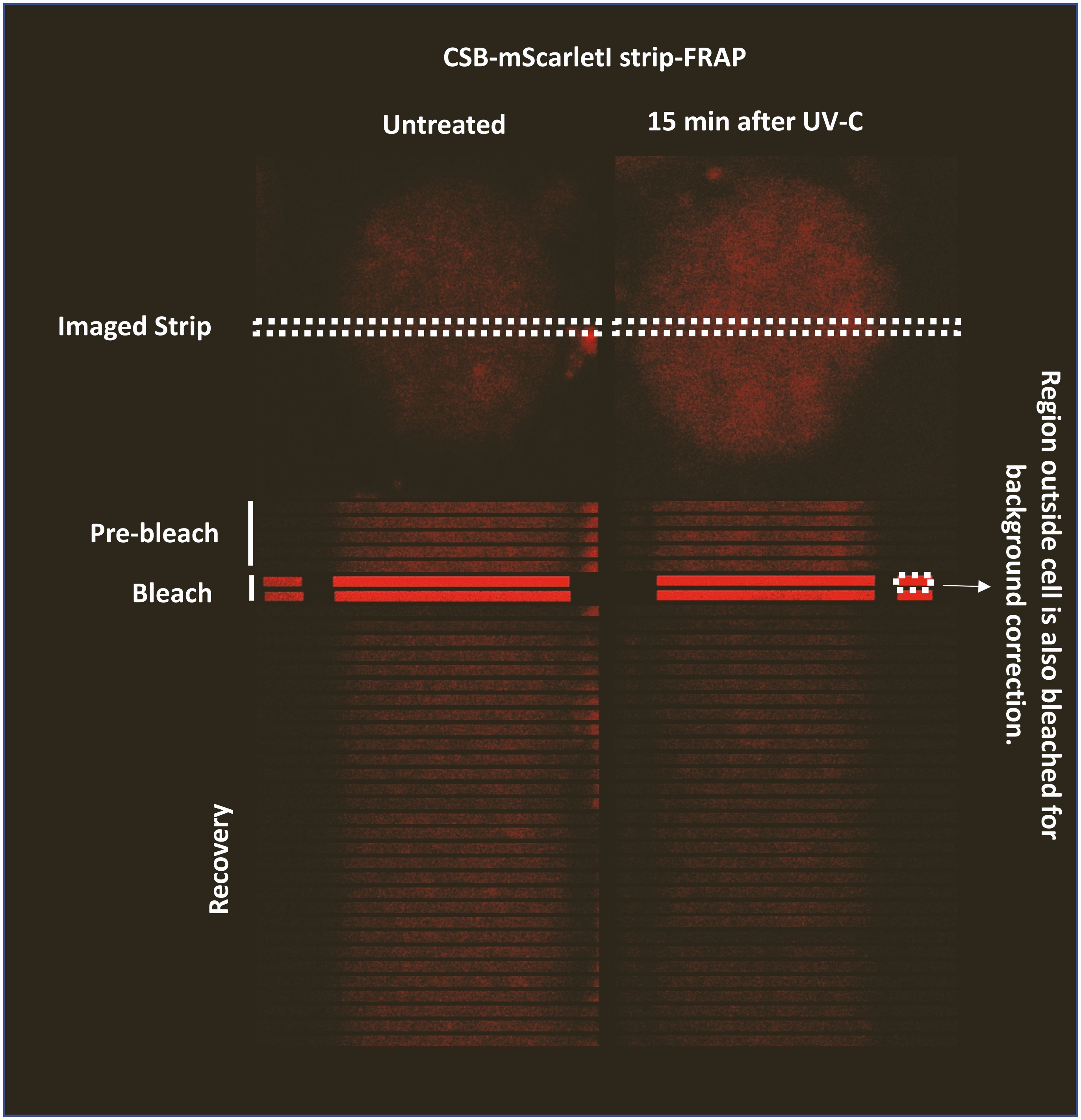

Supplemental Figure 3

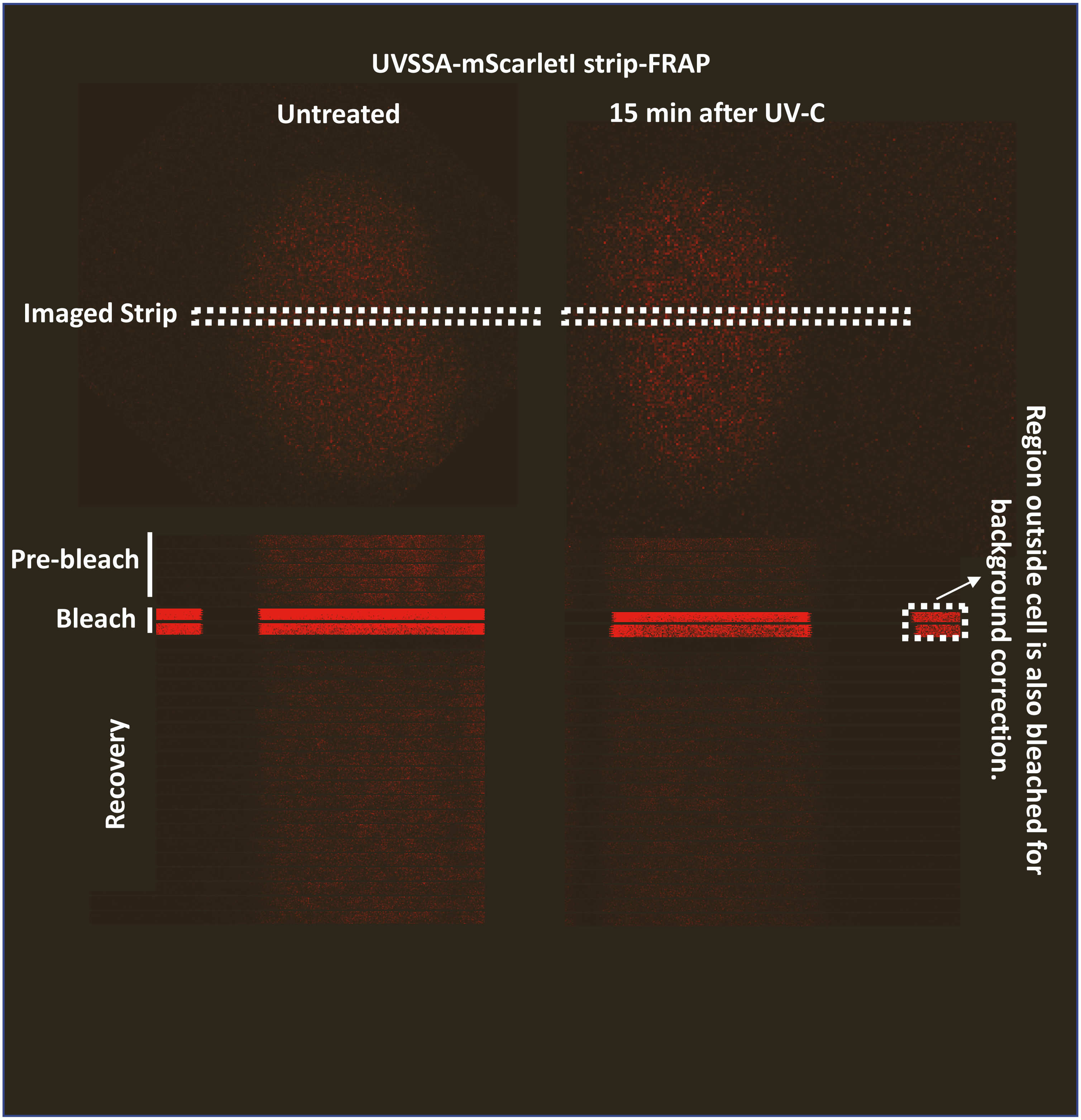

Supplementary Figure 4

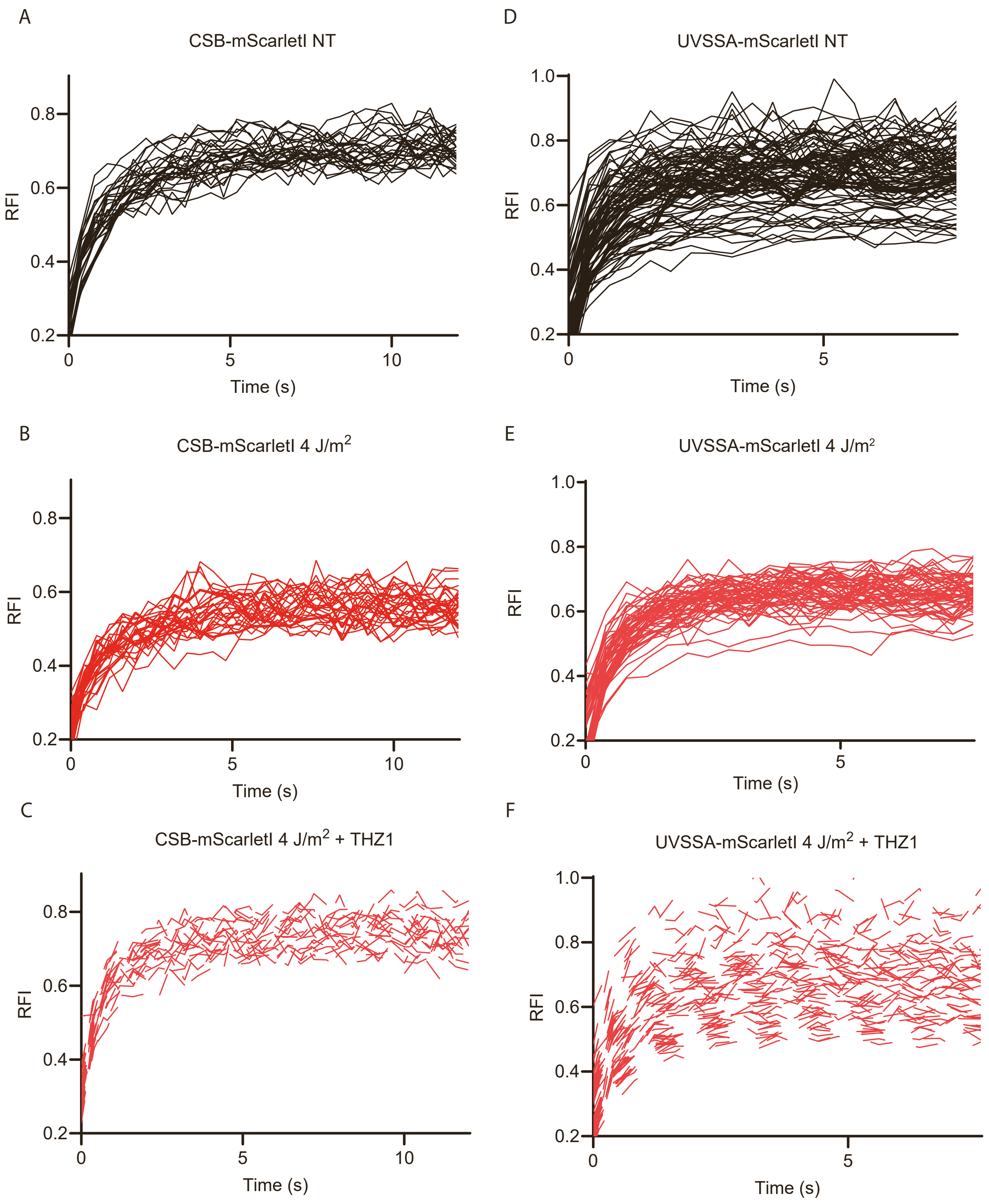

Supplementary Figure 1: Generation of TC-NER knock-out cells, related to figure 1.

(A) Cartoon of TC-NER mechanism. Elongating Pol II stalls at DNA damage, and lesion-stalled Pol II is recognized by CSB, which results in the assembly of the TC-NER complex including the recruitment of the CRL4^{CSA} ubiquitin E3 ligase that targets CSB and Pol II for ubiquitylation. Also UVSSA is recruited to the TC-NER complex, together with the de-ubiquitylating enzyme USP7 which thereby stabilizes CSB. UVSSA also recruits TFIIH which stimulates the unwinding of the DNA-damage site. Together with xeroderma pigmentosum group A (XPA) and replication protein A (RPA), TFIIH is responsible for the damage-verification and the recruitment of the XPF and XPG endonucleases that excise the DNA damage. Subsequently, the single-stranded gap generated by the excision is filled by DNA synthesis and sealed by DNA ligase, after which transcription can restart. Cartoon is created with BioRender.com.

(B) Histograms of mScarletI fluorescence levels in WT (red), CSB-mScarletI KI (Blue) and UVSSA-mScarletI cells (orange) as determined by flow cytometry analysis (FACS) of at least 4000 selected single and viable cells.

(C) Top panel: Western Blot analysis of HCT116 mScarletI-tagged CSB KI WT cells and HCT116 CRISPR/Cas9-mediated knock-out (KO) cells of the repair proteins CSA and XPA as indicated (top panel). Bottom panel: Sequencing results showing a homozygous 1 bp deletion in the UVSSA locus of UVSSA KO cells, resulting in a frame shift mutation.

(D) Relative colony survival of HCT116 mScarletI-tagged CSB KI WT and CSA, XPA, and UVSSA KO cells following exposure to indicated doses of UV-C. Plotted curves represent mean ± SEM. n=4 (WT, CSA KO, UVSSA KO), n=3 (XPA KO).

(E) Transcription restart after UV damage as determined by relative EU incorporation in indicated KO cells (HCT116 mScarletI-tagged CSB KI background), 2 or 24 hours after 8J/m^2 UV-C or mock treatment (NT) (left panel). Relative integrated density of UV-irradiated samples is normalized to mock-treated and set to 100. Black lines indicate average integrated density of, respectively n=1420, 747, 606, 708, 1253, 887, 540, 611, 851, 760, 384, 331 cells collected from 4 independent experiments. Right panel: Representative immunofluorescence images of EU incorporation in indicated HCT116 mScarletI-tagged CSB KI cells, 2 or 24 hours after 8J/m^2 UV-C or mock treatment (NT).

Supplementary Figure 2: Representative images of CSB-mScarletI strip-FRAP, related to figure 2H.

Representative images of the strip-FRAP procedure of CSB-mScarletI cells of untreated and UV (4 J/m^2) exposed cells. Top figure indicates entire cell, white square indicates the bleached and analyzed strip. Pre-bleach, bleach and post-bleach images of the strip are indicated below. Small bleached region outside the nucleus is used for background correction.

Supplementary Figure 3: Representative images of UVSSA-mScarletI strip-FRAP, related to figure 2G.

Representative images of the strip-FRAP procedure of CSB-mScarletI cells of untreated and UV (4 J/m^2) exposed cells. Top figure indicates entire cell, white square indicates the bleached and analyzed strip. Pre-bleach, bleach and post-bleach images of the strip are indicated below. Small bleached region outside the nucleus is used for background correction.

Supplementary Figure 4: Individual FRAP curves of single cells, related to figure 2 F and G.

(A-C): FRAP measurements of individual cells of FRAP measurements of CSB-mScarletI cells from experiment Fig. 2F main figures, either untreated (NT) **(A)**, exposed to 4 J/m^2 UV **(B)**, or pre-treated

with THZ1 before exposure to 4 J/m^2 UV **(C)**. **(D-F):** FRAP measurements of individual cells of FRAP measurements of UVSSA-mScarletI cells from experiment Fig. 2G main figures, either untreated (NT) **(D)**, exposed to 4 J/m^2 UV **(E)**, or pre-treated with THZ1 before exposure to 4 J/m^2 UV **(F)**.
